# Resveratrol Alleviates Levodopa-Induced Dyskinesia in Rats

**DOI:** 10.3389/fimmu.2021.683577

**Published:** 2021-06-25

**Authors:** Chang-Qing Zheng, Hong-Xia Fan, Xiao-Xian Li, Jing-Jie Li, Shuo Sheng, Feng Zhang

**Affiliations:** Key Laboratory of Basic Pharmacology of Ministry of Education and Joint International Research Laboratory of Ethnomedicine of Ministry of Education and Key Laboratory of Basic Pharmacology of Guizhou Province and Laboratory Animal Center, Zunyi Medical University, Zunyi, China

**Keywords:** L-DOPA, dyskinesia, neuroinflammation, resveratrol, Parkinson disease

## Abstract

Dyskinesia is a serious complication of Parkinson’s disease during levodopa (L-DOPA) treatment. The pathophysiology of L-DOPA-induced dyskinesia (LID) is complex and not fully illuminated. At present, treatment of dyskinesia is quite limited. Recent studies demonstrated neuroinflammation plays an important role in development of LID. Thus, inhibition of neuroinflammation might open a new avenue for LID treatment. Resveratrol (RES) is the most well-known polyphenolic stilbenoid and verified to possess a large variety of biological activities. DA neurotoxicity was assessed *via* behavior test and DA neuronal quantification. The movement disorders of dyskinesia were detected by the abnormal involuntary movements scores analysis. Effects of RES on glial cells-elicited neuroinflammation were also explored. Data showed that RES attenuated dyskinesia induced by L-DOPA without affecting L-DOPA’s anti-parkinsonian effects. Furthermore, RES generated neuroprotection against long term treatment of L-DOPA-induced DA neuronal damage. Meanwhile, RES reduced protein expression of dyskinesia molecular markers, ΔFOS B and ERK, in the striatum. Also, there was a strong negative correlation between DA system damage and ΔFOS B level in the striatum. In addition, RES inhibited microglia and astroglia activation in substantia nigra and subsequent inflammatory responses in the striatum during L-DOPA treatment. RES alleviates dyskinesia induced by L-DOPA and these beneficial effects are closely associated with protection against DA neuronal damage and inhibition of glial cells-mediated neuroinflammatory reactions.

## Introduction

Parkinson’s disease (PD) is the second most prevalent neurodegenerative disease in the world with an incidence of about 1%-2% (1, 2). The etiology of PD is closely related to environmental and genetic factors, which eventually leads to loss of dopamine (DA) neurons in the substantia nigra pars compacta (SNpc) and the subsequent DA neurotransmitter depletion in the striatum (3). Patients exhibit a range of clinical symptoms, such as slow movement, postural instability, resting tremor and muscle stiffness. DA replacement therapy based on levodopa (L-DOPA) is still a gold standard and widely used prescription therapy in clinic (4). L-DOPA mainly effectively improve the motor symptoms of PD. However, there is no evidence that L-DOPA therapy could prevent or delay the progression of DA neurodegeneration. Importantly, with the progression of the disease and the increase of cumulative L-DOPA exposure, patients tend to develop several severe drug side effects, such as dyskinesia (termed as L-DOPA-induced dyskinesia, LID) (5). Clinical symptoms of LID generally include chorea, dystonia and simple repetitive involuntary movement, which are one of the important causes of disability in patients with PD (6, 7).

The pathophysiology of LID is complex and not fully illuminated (8, 9). Studies indicated that intermittent administration of L-DOPA caused fluctuations in DA receptors in the striatum, leading to imbalance in glutamatergic, serotonergic and adrenergic system functions and signal transduction. In addition to neuronal mechanisms, non-neuronal mechanisms, such as glial cells-mediated neuroinflammation and angiogenesis, might contribute to the occurrence of LID (10, 11). Postmortem biochemical analysis presented the evidence of neuroinflammation in the brain of PD patients. A number of studies supported that repeated administration of L-DOPA aggravated neuroinflammation in the striatum (12, 13). Using compounds, such as Cannabidiol and Cannabinoid Compounds or Rho kinase inhibitor fasudil, that block different neuroinflammatory pathways could reduce dyskinesia movement disorders (14–16). In addition, endogenous anti-inflammatory agents, such as corticosterone, could apparently reduce the occurrence and development of LID. Also, IL-1β receptor antagonists attenuated LID *via* the decreased IL-1β production in the striatum (17). These investigations suggested that neuroinflammation was closely involved in the pathogenesis of LID.

To date, the only drug for the treatment of LID in PD patients is amantadine, a non-competitive antagonist of N-methyl-D-aspartate (NMDA) glutamate receptor. However, it could not be an ideal drug for long-term treatment of dyskinesia due to its tolerance and side effects (18). To find potential suitable alternatives for the treatment of dyskinesia is still an unmet clinical demand (19).

Resveratrol (trans-3, 4, 5-trihydroxystilbene, RES) is the most well-known polyphenolic stilbenoid, found in grapes, peanuts, mulberries and several other plants (20). RES has been verified to possess a large variety of biological activities, such as anti-oxidative, anti-aging, anti-inflammatory, anti-cancer and anti-microbial properties (21). The immunoregulatory role of RES was proposed almost 20 years ago. Thus, RES could play promising beneficial effects on the prevention of the progression of chronic diseases related to inflammation, such as cardiovascular diseases, diabetes and cancers (22–24). In addition, studies have shown that RES can cross blood-brain barrier (25), thus showing therapeutic potential in central nervous system (26). For example, RES (20 mg/kg) generated remarkable neuroprotective actions against neurodegenerative diseases in animal models (27–30). Moreover, studies have suggested that in PD animal models, RES conferred DA neuroprotection in substantia nigra *via* the activation of SIRT1 signaling (31). Although the underlying neuroprotective mechanism is not fully understood, it is closely associated with its anti-inflammatory and anti-oxidant properties (28).

Currently, DA neuronal loss is one of the pathological features of PD, which has been recognized to be an important risk factor for the development of LID (9). Based on the characteristics of neuroprotection and multi-acting targets of RES, role of RES on the pathogenesis of dyskinesia was explored in this study. Here, 6-hydroxydopamine (6-OHDA)-lesioned DA neuronal damage rat model was employed to investigate the activities of RES on L-DOPA’s therapeutic actions and side effects, such as LID, as well. Particularly, these findings might provide beneficial synergistic therapeutic avenues for PD.

## Methods

### Animals

Male Sprague-Dawley rats weighing 180-200 g were used. Rats were housed in a temperature (19-25°C) and humidity-controlled (40-70%) with a 12 h light/dark cycle and free access to autoclaved water and rat chow diet. All efforts were made to minimize their suffering. A total of 160 rats were randomly divided into 4 groups with 40 rats in each group, namely: 6-OHDA, 6-OHDA+RES, 6-OHDA+L-DOPA and 6-OHDA+L-DOPA +RES groups. Then, ten rats were sacrificed at each time point, in which 5 rats were applied for immunohistochemical staining and the other 5 rats were used for western blot analysis. All animal experiments were performed in accordance with National Institute of Health Guideline for the Animal Care and Use of Laboratory Animal and the protocols were approved by the institutional Animal Care and Use Committee at Zunyi Medical University (Zunyi, China).

### 6-OHDA Lesion and L-DOPA and RES Treatment

To create the 6-OHDA lesion, rats were anesthetized with sodium pentobarbital (60 mg/kg) and then immobilized in a stereotaxic frame to target the unilateral SNpc (coordinates AP-5.2, ML-2.1, DV-8.0) relative to bregma. A single 6-OHDA (5 μg/μL, diluted in normal saline containing 0.02% ascorbic acid; Sigma) solution was injected over 3 min with an infusion rate of 1 μL/min, followed by 3 min of equilibrium before retracting the needle. All rats were allowed to a 21-day recovery period before experiment start. Then, intraperitoneal injection of L-DOPA [L-DOPA methyl ester HCl (5 mg/kg) combined with benserazide HCl (2.5 mg/kg) diluted in 0.9% saline; Sigma] and intragastrical administration of RES (20 mg/kg, diluted in 0.1% carboxymethylcellulose sodium aqueous solution; Sigma) were performed daily.

### Rotarod Test

The rotarod treadmills were made of a rotating spindle (7.6 cm diameter) and 4 individual compartments able to simultaneously test four rats. Before the experiment, rats were subjected to two days of adaptive training, and the speed was fixed at 12 rpm. In the formal test, the acceleration parameter was selected to be 4.0-40 rpm for the experiment. Each rat was tested 3 times with an interval of 30 min. Finally, the average stay time of 3 times in the rod of each rat was statistically analyzed.

### Stepping Test

The stepping test was employed to measure rat forelimb akinesia. Rats with unilateral DA depletion of more than 80% performed poorly in this test (32). L-DOPA treatment could greatly improve this defect. Therefore, when adjuvant drugs were used in combination with L-DOPA, stepping test was also used to determine whether additional adjuvant drugs affected the efficacy of L-DOPA (33, 34). In detail, the stepping test was performed 30 min after L-DOPA treatment. The two forepaws were tested alternately and each forepaw was tested 3 times. Rats were held by the investigator immobilizing the hind legs with one hand and one forepaw not to be monitored with the other, so that the weight of rat fell completely on the test forelimb. Then, rats were moved across the table at a speed of 90 cm/5 s, during which an additional investigator recorded the number of adjustment steps. Data were presented as mean percent intact stepping, where the sum of the total steps with the lesioned forepaw was divided by the total steps with the unlesioned forepaw multiplied by 100. Lower percent intact scores indicated greater forelimb akinesia.

### Assessment of Abnormal Involuntary Movements (AIMs)

L-DOPA induced AIMs scores were evaluated on 7, 21, 42 and 84 days after L-DOPA treatment. Dyskinesia behavior changes were assessed for 1min every 30-min interval over a 120-min period. The following three subtypes of AIMs: Axial, Limb and Orofacial AIMs scores were evaluated. Each subtype AIM was scored based on a frequency from 0 to 4 (0=absent, 1=intermittently present for 50% of the observation period, 2=intermittently present for > 50% of the observation period, 3=interruptable and present through the entire rating period, 4=uninterruptable and through the entire rating period). Through the AIMs test, the severity of dyskinesia in each rat was quantified. The total AIM scores were obtained by summing each AIMs subtype score from each 30-min observation period.

### Western Blot Analysis

After animals were deeply anesthetized, brains were quickly dissected and separated on ice and the separated midbrain and striatum tissues were frozen at -80°C. The frozen tissue was homogenized with lysis buffer (including protease inhibitor and phosphate protease inhibitor) and cracked on ice. The supernatant was collected after centrifugation for 10 min at 4°C. Protein concentrations were detected by BCA assay kit. An equal of amount of protein for each sample was loaded onto an sodium dodecyl sulfate polyacrylamide electrophoresis gel (10%). Proteins separated by gel electrophoresis were electro-transferred onto a 0.45 mm polvinylidene difluoride membranes. The membranes were blocked with 5% skimmed milk, and then incubated with the following primary antibodies at 4°C overnight: rabbit anti-TH (1:2000; Proteintech), rabbit anti-IL-1β (1:1000; Proteintech), rabbit anti-TNF-α (1:1000; Proteintech), rabbit anti-COX-2 (1:1000; Abcam), rabbit anti-Inos (1:1000; Proteintech), rabbit anti-p-ERK (Thr202/204) (1:1000; Affinity), rabbit anti-ERK (1:1000; Proteintech), rabbit anti-ΔFOS B (1:1000; HuaAn Biotechology), rabbit anti-GAPDH (1:2000; Servivebio), rabbit anti-β-actin (1:2000; Servivebio). After washing, bound antibodies were detected with HRP-conjugated secondary anti-rabbit antibody (1:3000; Proteintech). The blots were developed with enhanced chemiluminescence antoradiography (ECL kit) and quantified with the software Quantity one.

### Immunohistochemical Staining

Rats were perfused intracardially with PBS and subsequently fixed with 4% paraformaldehyde. After tissue was dehydrated and embedded in paraffin, a total of 5-μm section was cut and collected. Antigens were repaired *via* high temperature and pressure antigen repair using citrate buffer (pH=6). Tissue was cooled to room temperature and incubated in goat serum for 30 min, and incubated with the following primary antibodies at 4 overnight: rabbit anti-TH (1:500; Abcam), rabbit anti-IBA-1 (1:300; Abcam), rabbit anti-GFAP (1:500; Proteintech), rabbit anti-ΔFOS B (1:100; HuaAn Biotechology). For immunohistochemical staining, slides were incubated with biotinylated secondary antibody at 37°C for 15 min and visualized with avidin horseradish enzyme at 37°C for 20 min. Slices were visualized through DAB kit. For immunofluorescence staining, the second antibodies were anti-rabbit IgG Alexa Flour 488 (1:800; Abcam). Quantification of TH-positive neurons was performed through visually counting the number of TH-positive neuronal cell bodies blindly by two investigators. The images were recorded with a charge-coupled device camera and operated with the MetaMorph software. The results were obtained from the average. The mean value for the number of TH-positive neurons in substantia nigra was then deduced by averaging the counts of six sections for each rat.

### Statistics

Data were expressed as mean ± standard error of the mean (SEM). Statistical significance was analyzed by one- or two-way analysis of variance (ANOVA) using GraphPad Prism software (GraphPad Software Inc., San Diego, CA, USA). When ANOVA showed significant difference, pairwise comparisons between means were analyzed by Bonferroni’s *post hoc t-*test with correction. A value of *p*<0.05 was considered statistically significant. In addition, the correlation between DA system damage and ΔFOS B level was tested by Pearson correlation analysis.

## Results

### RES Alleviated Dyskinesia Induced by L-DOPA

**Figure 1:**
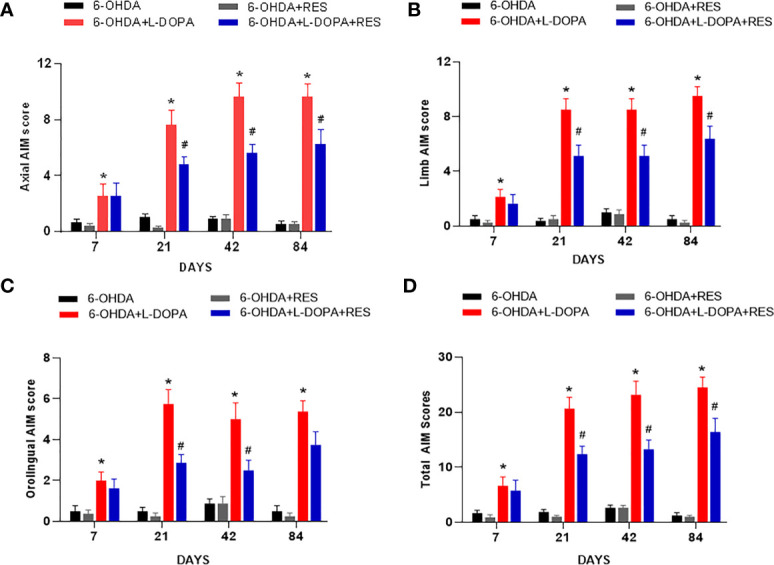
RES alleviated dyskinesia induced by L-DOPA. L-DOPA- induced behavior changes of axial **(A)**, limb **(B)**, orafacial **(C)** and total AIM scores **(D)** were evaluated 7, 21, 42 and 84 days after L-DOPA administration, respectively. Data were expressed as mean ± SEM from 10 rats. *P < 0.05 compared with 6-OHDA group; ^#^ *P* < 0.05 compared with 6-OHDA+L-DOPA group.

Rat Dyskinesia behavior dysfunction induced by L-DOPA was evaluated by AIM scores 7, 21, 42 and 84 days after L-DOPA application. As shown in **Figure 1**, L-DOPA aggravated the behavior dysfunction of axial, limb, orafacial and total AIM scores beginning from L-DOPA treatment for 7 days. However, RES attenuated L-DOPA-induced AIM scores aggravation from RES administration for 21 days.

### RES Didn’t Affect L-DOPA’s Anti-PD Efficacy

**Figure 2:**
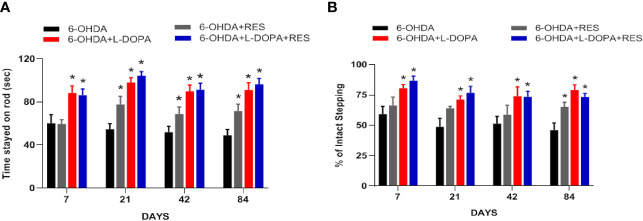
RES didn’t affect L-DOPA’s anti-PD efficacy. The beneficial effects generated by co-treatment of RES with L-DOPA on 6-OHDA-induced rat behavior changes were analyzed by rotarod test **(A)**. The time rat stayed on the rod was recorded. The effects of RES together with L-DOPA on forelimb stepping changes were determined *via* the stepping test to further evaluate whether RES affected L-DOPA’s efficacy **(B)**. Data were expressed as mean ± SEM from 10 rats. *P < 0.05 compared with 6-OHDA group.

L-DOPA’s efficacy of anti-PD motor dysfunction was assessed by rotarod test. As shown in **Figure 2A**, compared with 6-OHDA group, L-DOPA attenuate 6-OHDA-induced decrease of the time rat stayed on rod from L-DOPA treatment for 7 days, while RES attenuated this motor dysfunction from RES treatment for 21 days. In addition, compared with L-DOPA or RES treatment, no further changes were shown in L-DOPA combined with RES treatment. On the other hand, to evaluate whether RES affected L-DOPA’s anti-PD efficacy, rat motor performance was analyzed by stepping test. As shown in **Figure 2B**, L-DOPA reversed 6-OHDA-induced stepping deficits from L-DOPA treatment for 7 days. Moreover, RES combined with L-DOPA preserved L-DOPA-generated this beneficial effect. These results showed that RES had no negative effects on the improvement of L-DOPA against PD motor dysfunction.

### Effects of RES Combined With L-DOPA on 6-OHDA-Induced DA Neurotoxicity

**Figure 3:**
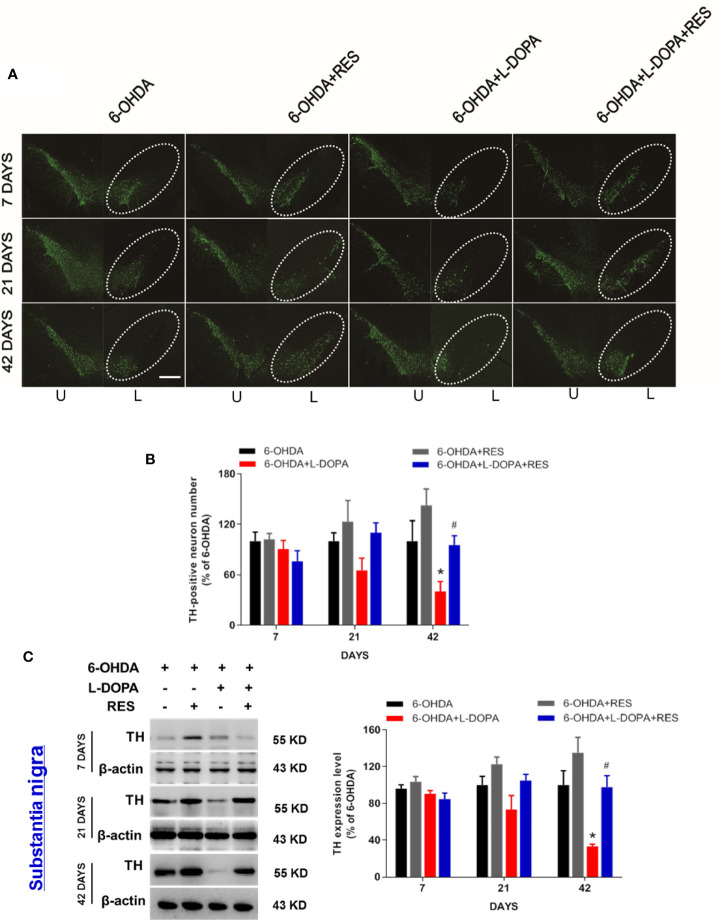
Effects of RES combined with L-DOPA on 6-OHDA-induced DA neurotoxicity. 7, 21 and 42 days after L-DOPA treatment, rats were sacrificed and brains were collected, respectively. DA neurons in substantia nigra were recognized and immunostained with an anti-TH antibody **(A)**. The “ellipse” presented the area of substantia nigra. DA neuronal loss in substantia nigra was analyzed *via* the quantification of TH-positive neurons. U, unlesioned side; L, lesioned side. **(B)**. Scale bar=100 µm. TH protein level was determined by western blotting **(C)**. The densitometry values of TH were detected and normalized to β-actin of the same group. Then, data were normalized and calculated as a percentage of each respective 6-OHDA group. Data were expressed as mean ± SEM from 5 rats. *P < 0.05 compared with 6-OHDA group; ^#^ *P* < 0.05 compared with 6-OHDA+L-DOPA group.

Next, the effects of RES combined with L-DOPA on 6-OHDA-induced DA neuronal loss were investigated. As shown in **Figures 3A, B**, after 7 and 21 days of L-DOPA administration, there was no significant difference of DA neuronal number between the 6-OHDA group and the other groups. After 42 days of L-DOPA treatment, compare with the 6-OHDA group, L-DOPA caused more DA neuronal damage. However, RES exerted neuroprotection against L-DOPA-induced DA neuronal damage. Consistent with DA neurons quantification analysis, TH (DA neuron marker) protein expression detection also demonstrated that RES attenuated L-DOPA-induced DA neurotoxicity (**Figure 3C**). To investigate whether RES and L-DOPA affected DA neuronal survival on the unlesioned side of substantia nigra, the effects of L-DOPA and RES on contralateral unlesioned side was determined by TH-positive neuron quantification and TH protein expression detection. As shown in **Supplemental Figure 1**, no significant difference of TH-positive neuron number and TH protein expression in contralateral unlesioned side of substantia nigra among 6-OHDA, 6-OHDA+RES, 6-OHDA+RES+L-DOPA and 6-OHDA+RES+L-DOPA groups after 42 days of L-DOPA treatment was discerned.

### RES Reduced the Protein Expression of Dyskinesia Molecular Markers ΔFOS B and p-ERK in Rat Striatum

**Figure 4:**
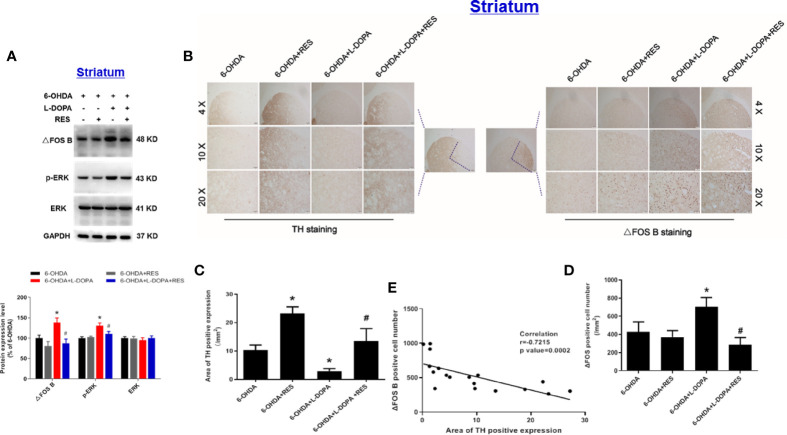
Analysis of the correlation between DA neuronal damage and dyskinesia marker ΔFOS B level in the striatum of rats with LID. After 42 days of L-DOPA treatment, the expression levels of ΔFOS B, phosphorylated-ERK (p-ERK) and ERK proteins in the striatum of rats were analyzed by western blotting. The densitometry values of TH, phosphorylated ERK (p-ERK) and ERK were detected and normalized to GAPDH of the same group. Then, data were normalized and calculated as a percentage of each respective 6-OHDA group **(A)**. Next, the expression of TH from DA projection fiber and ΔFOS B in the striatum of rats were detected by immunohistochemistry staining **(B)**. Area of TH-positive expression was measured **(C)**. ΔFOS B-positive cell number was quantified **(D)**. The correlation between the expression of TH and ΔFOS B was analyzed **(E)**. Data were expressed as mean ± SEM from 5 rats. *P < 0.05 compared with 6-OHDA group; ^#^ *P* < 0.05 compared with 6-OHDA+L-DOPA group.

After 42 days of L-DOPA administration, the effects of L-DOPA or RES on protein expression of ΔFOS B, phosphorylated-ERK (p-ERK) and total ERK in the ipsilateral striatum of rats were detected. As shown in **Figure 4A**, compared with 6-OHDA group, L-DOPA increased the protein expression of ΔFOS B and p-ERK, which was reduced by RES treatment. Then, analysis of the correlation between DA neuronal damage and ΔFOS B level in the striatum of rats with LID was performed. First, the expression of ΔFOS B and TH (DA projection fiber) in the striatum of rats were measured. As shown in **Figures 4B–D**, compared with 6-OHDA group, L-DOPA decreased TH expression and increased ΔFOS B expression in the striatum. After RES treatment, RES reversed L-DOPA-induced changes of TH and ΔFOS B. Then, the correlation between TH and ΔFOS B level during LID was further analyzed. As shown in **Figure 4E**, there was a strong negative correlation between DA neuronal damage and ΔFOS B level in the striatum.

### RES Inhibited Neuroinflammatory Reactions Induced by L-DOPA

**Figure 5:**
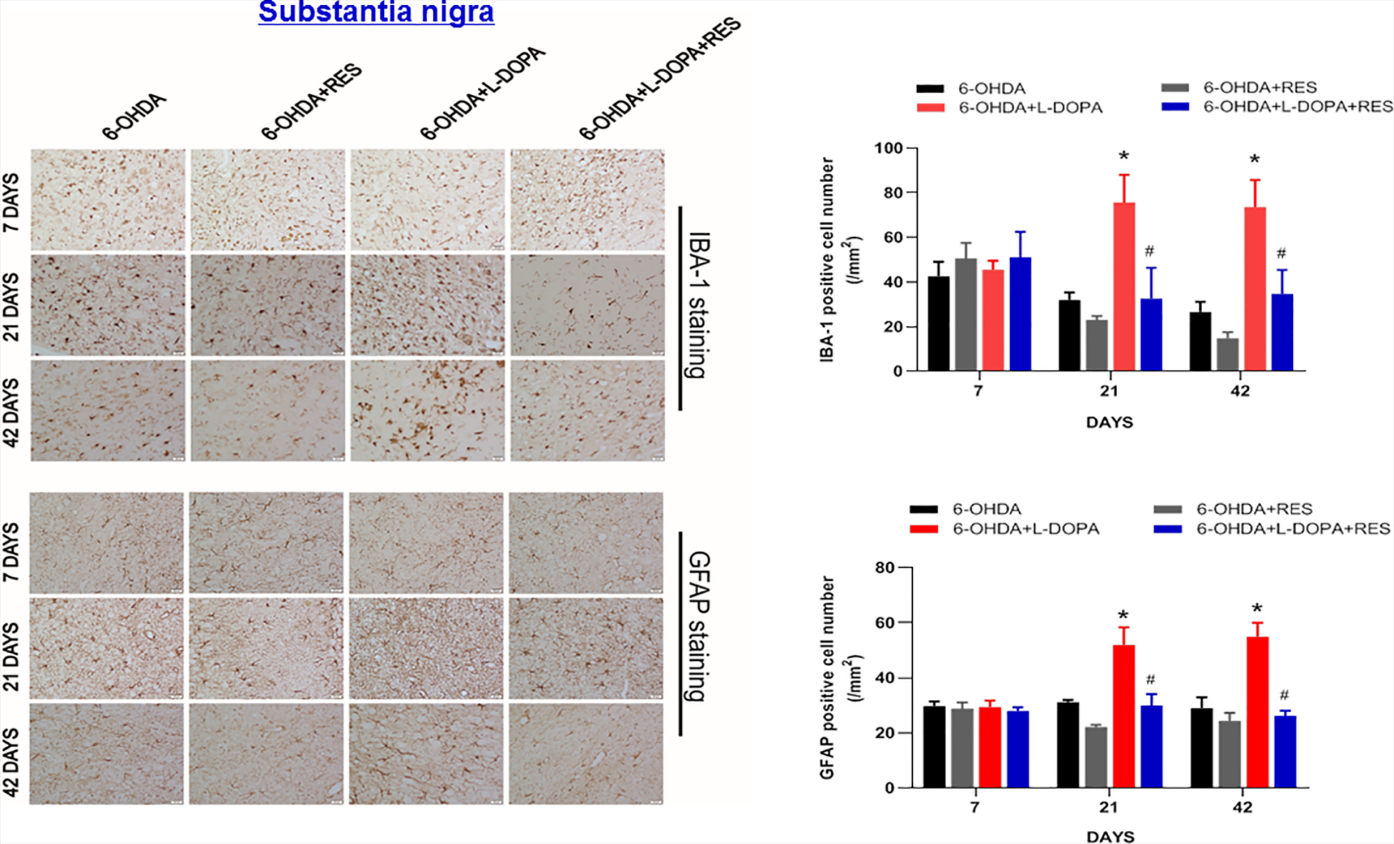
Effects of RES on activation of glial cells in substantia nigra of rats with LID. Rat brains were sectioned and microglia and astroglia were immunostained with anti-GFAP and IBA-1 antibodies 7, 21, and 42 days after L-DOPA administration, respectively. The number analysis of IBA-1-positive microglia and GFAP-positive astroglia in substantia nigra from 3 evenly spaced brain sections of each rat was performed.Data were expressed as mean ± SEM from 5 rats and calculated as percentage of 6-OHDA values. *P < 0.05 compared with 6-OHDA group; ^#^ *P* < 0.05 compared with 6-OHDA+L-DOPA group.

**Figure 6:**
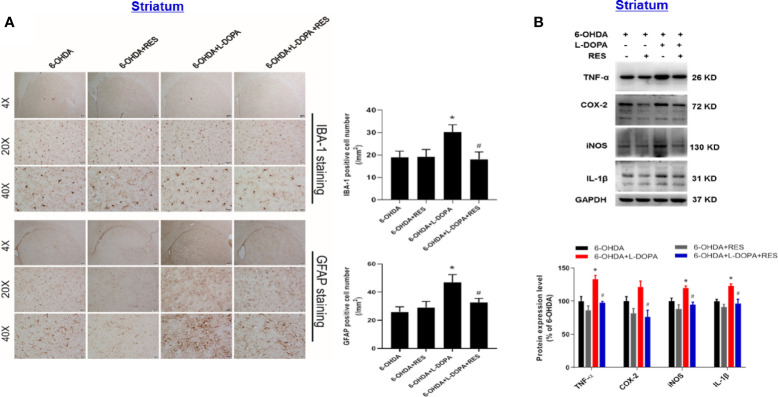
Effects of RES on inflammatory responses in the striatum of rats with LID. Rat striatum tissue were sectioned and immunostained with anti-GFAP and IBA-1 antibodies 42 days after L-DOPA administration. The number analysis of IBA-1-positive microglia and GFAP-positive astroglia in the striatum from 3 evenly spaced brain sections of each rat was determined **(A)**. The protein levels of pro-inflammatory mediators, such as TNF-α, iNOS, COX-2 and IL-1β, in the striatum 42 days after L-DOPA administration were detected by western blot assay **(B)**. The densitometry values of TNF-α, iNOS, COX-2 and IL-1β were detected and normalized to GAPDH of the same group. Then, data were normalized and calculated as a percentage of each respective 6-OHDA group. Data were expressed as mean ± SEM from 5 rats. *P < 0.05 compared with 6-OHDA group; ^#^ *P* < 0.05 compared with 6-OHDA+L-DOPA group.

Since neuroinflammation was considered to be one of the important causes of LID, the role of glial cells-mediated neuroinflammation in RES-attenuated LID was explored. As shown in **Figure 5**, compared with 6-OHDA group, L-DOPA induced microglia (IBA-1) and astroglia (GFAP) activation in the substantia nigra from L-DOPA treatment for 21 days. However, RES inhibited L-DOPA-induced glial cells activation. To further confirm the inactivation of glial cells mediated by RES, the effects of RES on glial cells-elicited inflammatory responses in the striatum were determined. As shown in **Figure 6A**, similar results were present that RES suppressed microglia and astroglia activation induced by L-DOPA in the striatum. Furtherly, the protein expression of pro-inflammatory mediators in rat striatum were detected. As shown in **Figure 6B**, L-DOPA increased TNF-α, COX-2, iNOS and IL-1β protein expressions, which could be reduced by RES treatment.

## Discussion

The purpose of this study was to determine whether the “multi-target drug” RES alleviated dyskinesia induced by L-DOPA. Results clearly indicated RES attenuated LID without affecting L-DOPA’s anti-parkinsonian effects. Furthermore, RES generated neuroprotection against long term treatment of L-DOPA-induced DA neuronal damage. Meanwhile, RES reduced the protein expression of dyskinesia molecular markers, ΔFOS B and ERK, in the striatum. Also, there was a strong negative correlation between DA system damage and ΔFOS B level in the striatum. In addition, RES inhibited microglia and astroglia activation in substantia nigra and subsequent inflammatory responses in the striatum during L-DOPA-treated PD. Collectively, these results suggested that RES attenuated LID through protection against DA neuronal damage and inhibition of glial cells-mediated neuroinflammatory reactions.

Nowadays, clinical studies indicated that the course of PD was a risk factor for the onset of dyskinesia (35), and the loss of DA neurons might be directly related to the severity of LID. Moreover, the rapid increase of exogenous DA caused by L-DOPA administration and the volatile stimulation of DA receptor was an important cause of dyskinesia. The remaining DA neurons in PD were an important source of endogenous DA. With the aggravation of the course of PD and the increase of the loss of DA neurons, endogenous DA was not sufficient to cushion the fluctuation of neurotransmitters caused by exogenous DA. In addition, L-DOPA could produce cytotoxic reactive oxygen species through oxidative metabolism of DA or autoxidation (36). Long-term administration of L-DOPA in 6-OHDA-damaged rats might lead to the accumulation and metabolism of extracellular DA in the striatum, which could magnify the pre-existing oxidative and pro-inflammatory environment, thus promoting the process of DA neurodegeneration. Until now, the neuroprotective effects of RES have been widely proved. In this study, it has been proved that RES protected DA neurons from L-DOPA-induced neurotoxicity. At the same time, the correlation analysis of striatal DA projecting fibers and ΔFOS B demonstrated that the loss of DA neurons was directly related to the severity of LID.

At present, the pathogenesis of LID is very complicated. As mentioned earlier, the decrease of DA projecting fibers in the striatum of PD led to rapid changes in the concentration of DA neurotransmitters after L-DOPA treatment, resulting in changes in the conduction function of basal ganglia. A number of studies showed that the increased activity of D1R led to changes in a series of downstream molecular levels, such as phosphorylation of cyclic adenosine monophosphate phosphorylated protein-32 (DARPP-32) (37), ERK1/2 and increased expression of early gene proteins, such as ΔFOS B (38, 39). Here, this study exhibited that RES could reduce the expression of representative molecules, such as ERK and ΔFOS B protein, to ameliorate LID.

Although neuroinflammation is verified to contribute to the pathogenesis of PD, recent studies have confirmed that neuroinflammation also plays an important role in LID (10). The role of neuroinflammation in LID is mainly mediated by glial cells and cytokines. Also, the effects of different administration of L-DOPA on dyskinesia and neuroinflammation were compared. Evidence indicated that there was no dyskinesia or inflammation in the continuous L-DOPA administration group, while intermittent L-DOPA treatment could lead to dyskinesia, glial cells activation and up-regulation of inflammatory factors in the striatum (40). Furthermore, intermittent L-DOPA administration pretreated with LPS aggravated the severity of neuroinflammation and dyskinesia in rats (40). These findings suggested that the inflammatory state in the striatum was closely related to the severity of dyskinesia. Additionally, microglia activation is an important manifestation of neuroinflammation and participates in the secretion of a variety of pro-inflammatory factors. For example, TNF-α was one of the most representative inflammatory factors. Existing studies demonstrated that TNF-α might be involved in the occurrence and development of LID through the following pathways: 1) TNF-α could activate a variety of intracellular signal transduction pathways, including NF-κB and MAPK signal pathway (41, 42), and further aggravated neuroinflammation; 2) TNF-α stimulated the release of glutamate from microglia and increased the extracellular concentration of excitatory neurotransmitter glutamate in the striatum (43); 3) Since angiogenesis was considered to be a risk factor for dyskinesia (13), reducing the expression of TNF-α inhibited angiogenesis to further attenuate dyskinesia (44). Emerging evidence indicated that the increased expression of various pro-inflammatory factors in the striatum of rats with dyskinesia and the decrease of inflammatory mediators, such as TNF-α (13), COX-2 (38, 45), IL-1β (17) and iNOS (12, 46), could reduce the severity of LID. On the other hand, the activation of astroglia is also closely associated with the occurrence of LID. First, astroglia are well-known to uptake and release L-DOPA (47, 48), and convert L-DOPA into DA (49), indicating that astroglia might be an important source of exogenous DA in the striatum. Second, monoamine oxidase in astroglia metabolizes DA, to produce toxic reactive oxygen species and DA metabolites (50). These products could further activate microglia and promote the secretion of pro-inflammatory factors in microglia. Besides, in animal models of PD, vascular endothelial growth factor mainly expressed in astroglia is recognized to be involved in the pathophysiology of LID (11). In this study, L-DOPA induced microglia and astroglia activation from L-DOPA treatment for 21 days. Similar phenomenon was exhibited that L-DOPA induced microglia activation and subsequent neuroinflammatory reactions in rat model after L-DOPA treatment for 36 days (51). However, current studies also demonstrated that L-DOPA reduced glial cells activation and neuroinflammatory responses (52). Thus, the mechanisms underlying the role of glial cells-mediated neuroinflammation on L-DOPA-treated PD were a quite interesting area to be worth further investigation. On the other hand, combined with L-DOPA administration, RES suppressed microglia and astroglia activation-mediated neuroinflammatory responses during LID. These data suggested that inhibition of neuroinflammation participate in RES-alleviated dyskinesia induced by L-DOPA.

Taken together, this study revealed the role of anti-inflammatory therapy on improving the occurrence of dyskinesia upon L-DOPA treatment. Results demonstrated that RES combined with L-DOPA could reduce further loss of DA neurons and attenuated the severity of LID. However, this study only focuses on the therapeutic effect of RES on dyskinesia induced by L-DOPA. Next, to further investigate whether anti-dyskinetic effects of RES are reversible after withdrawal or prior to L-DOPA warrants future investigation (53).

## Conclusions

RES alleviated dyskinesia induced by L-DOPA and these beneficial effects were closely associated with protection of DA neuronal damage and inhibition of glial cells-mediated neuroinflammatory responses.

## Data Availability Statement

The original contributions presented in the study are included in the article/**Supplementary Material**. Further inquiries can be directed to the corresponding author.

## Ethics Statement

All animal experiments were performed in accordance with National Institute of Health Guideline for the Animal Care and Use of Laboratory Animal and the protocols were approved by the institutional Animal Care and Use Committee at Zunyi Medical University (Zunyi, China).

## Author Contributions

C-QZ participated in the design, execution, data analysis and writing of the manuscript. H-XF, X-XL, J-JL, and SS participated in the revision of the article, and FZ participated in the design and revision of the article. All authors contributed to the article and approved the submitted version.

## Funding

This study was supported by the National Natural Science Foundation of China (No. 81760658), the foundation for High-level Innovative Talents of Guizhou Province (No. 20164027), the Innovation Research Group project of Education Department of Guizhou Province (No. 2016038) and the foundation for Excellent Young Talents of Zunyi Medical University (No.201603).

## Conflict of Interest

The authors declare that the research was conducted in the absence of any commercial or financial relationships that could be construed as a potential conflict of interest.

## Supplementary Material

Supplementary Figure 1 Effects of RES and L-DOPA on DA neuronal survival in the unlesioned side of substantia nigra. After 42 days of L-DOPA treatment, rats were sacrificed and brains were collected, respectively. DA neurons in substantia nigra were recognized and immunostained with an anti-TH antibody. DA neuronal survival in the unlesioned side of substantia nigra was analyzed *via* the quantification of TH-positive neurons **(A)** and the expression of TH protein **(B)**. Scale bar=100 µm. The densitometry values of TH protein were detected and normalized to GAPDH of the same group. Then, data were normalized and calculated as a percentage of each respective 6-OHDA group. Data were expressed as mean ± SEM from 5 rats.

## References

[1] Mayeux R. Epidemiology of Neurodegeneration. Annu Rev Neurosci (2003) 26(1):81. 10.1146/annurev.neuro.26.043002.094919 12574495

[2] Alves G Forsaa EB Pedersen KF Gjerstad MD Larsen JP. Epidemiology of Parkinson’s Disease. J Neurol (2008) 255(s5):18–32. 10.1007/s00415-008-5004-3 18787879

[3] Schapira AH Jenner P. Etiology and Pathogenesis of Parkinson’s Disease. Mov Disord (2011) 26(6):1049–55. 10.1002/mds.23732 21626550

[4] Olanow C Stern M Sethi K. The Scientific and Clinical Basis for the Treatment of Parkinson Disease. Neurology (2009) 72:S1–136. 10.1212/WNL.0b013e3181a1d44c 19470958

[5] Warren Olanow C Kieburtz K Rascol O Poewe W Schapira A Emre M. Factors Predictive of the Development of Levodopa-Induced Dyskinesia and Wearing-Off in Parkinson’s Disease. Mov Disord (2013) 28(8):1064–71. 10.1002/mds.25364 23630119

[6] Van Laar T. Levodopa-Induced Response Fluctuations in Patients With Parkinson’s Disease: Strategies for Management. CNS Drugs (2003) 17(7):475–89. 10.2165/00023210-200317070-00002 12751918

[7] Calabresi P Di Filippo M Ghiglieri V Tambasco N Picconi B. Levodopa-Induced Dyskinesias in Patients With Parkinson’s Disease: Filling the Bench-to-Bedside Gap. Lancet Neurol (2010) 9(11):1106–17. 10.1016/s1474-4422(10)70218-0 20880751

[8] Huot P Johnston TH Koprich JB Fox SH Brotchie JM. The Pharmacology of l-DOPA-Induced Dyskinesia in Parkinson’s Disease. Pharmacol Rev (2013) 65(1):171–222. 10.1124/pr.111.005678 23319549

[9] Bastide M Meissner W Picconi B Fasano S Fernagut P Feyder M. Pathophysiology of L-DOPA-Induced Motor and Non-Motor Complications in Parkinson’s Disease. Prog Neurobiol (2015) 132:96–168. 10.1016/j.pneurobio.2015.07.002 26209473

[10] Pisanu A Boi L Mulas G Spiga S Fenu S Carta A. Neuroinflammation in L-DOPA-Induced Dyskinesia: Beyond the Immune Function. J Neural Transm (2018) 125(8):1287–97. 10.1007/s00702-018-1874-4 29541852

[11] Ohlin K Francardo V Lindgren H Sillivan S O’Sullivan S Luksik A. Vascular Endothelial Growth Factor Is Upregulated by L-DOPA in the Parkinsonian Brain: Implications for the Development of Dyskinesia. Brain (2011) 134:2339–57. 10.1093/brain/awr165 PMC3155708 21771855

[12] Bortolanza M Cavalcanti-Kiwiatkoski R Padovan-Neto F da-Silva C Mitkovski M Raisman-Vozari R. Glial Activation Is Associated With l-DOPA Induced Dyskinesia and Blocked by a Nitric Oxide Synthase Inhibitor in a Rat Model of Parkinson’s Disease. Neurobiol Dis (2015) 73:377–87. 10.1016/j.nbd.2014.10.017 25447229

[13] Boi L Pisanu A Greig N Scerba M Tweedie D Mulas G. Immunomodulatory Drugs Alleviate L-Dopa-Induced Dyskinesia in a Rat Model of Parkinson’s Disease. Mov Disord Off J Mov Disord Soc (2019) 34(12):1818–30. 10.1002/mds.27799 PMC11719776 31335998

[14] Junior N Dos-Santos-Pereira M Guimarães F Del Bel E. Cannabidiol and Cannabinoid Compounds as Potential Strategies for Treating Parkinson’s Disease and L-DOPA-Induced Dyskinesia. Neurotoxic Res (2020) 37(1):12–29. 10.1007/s12640-019-00109-8 31637586

[15] Lopez-Lopez A Labandeira C Labandeira-Garcia J Muñoz A. Rho Kinase Inhibitor Fasudil Reduces l-DOPA-Induced Dyskinesia in a Rat Model of Parkinson’s Disease. Br J Pharmacol (2020) 177(24):5622–41. 10.1111/bph.15275 PMC7707090 32986850

[16] Teema A Zaitone S Moustafa YJN. Ibuprofen or Piroxicam Protects Nigral Neurons and Delays the Development of L-Dopa Induced Dyskinesia in Rats With Experimental Parkinsonism: Influence on Angiogenesis. Neuropharmcology (2016) 107:432–50. 10.1016/j.neuropharm.2016.03.034 27016022

[17] Barnum C Eskow K Dupre K Blandino P Deak T Bishop C. Exogenous Corticosterone Reduces L-DOPA-Induced Dyskinesia in the Hemi-Parkinsonian Rat: Role for Interleukin-1beta. Neuroscience (2008) 156(1):30–41. 10.1016/j.neuroscience.2008.07.016 18687386 PMC2615135

[18] Thomas A Iacono D Luciano A Armellino K Di Iorio A Onofrj M. Duration of Amantadine Benefit on Dyskinesia of Severe Parkinson’s Disease. J Neurol Neurosurg Psychiatry (2004) 75(1):141–3. PMC1757492 14707325

[19] Meissner WG Frasier M Gasser T Goetz CG Bezard E. Priorities in Parkinson’s Disease Research. Nat Rev Drug Discov (2011) 10(5):377–93. 10.1038/nrd3430 21532567

[20] Ahmadi Z Mohammadinejad R Ashrafizadeh M. Drug Delivery Systems for Resveratrol, a Non-Flavonoid Polyphenol: Emerging Evidence in Last Decades. J Drug Deliv Sci Technol (2019) 51:591–604. 10.1016/j.jddst.2019.03.017

[21] Britton R Kovoor C Brown K. Direct Molecular Targets of Resveratrol: Identifying Key Interactions to Unlock Complex Mechanisms. Ann New York Acad Sci (2015) 1348(1):124–33. 10.1111/nyas.12796 26099829

[22] Gal R Praksch D Kenyeres P Rabai M Toth K Halmosi R. Hemorheological Alterations in Patients With Heart Failure With Reduced Ejection Fraction Treated by Resveratrol. Cardiovascular Therapeutics (2020) 7262474:1–8. 10.1155/2020/7262474 PMC7350166 32695229

[23] Vervandier-Fasseur D Latruffe N. The Potential Use of Resveratrol for Cancer Prevention. Molecules (2019) 24(24):4506. 10.3390/molecules24244506 PMC6943596 31835371

[24] Sinha D Sarkar N Biswas J Bishayee A. Resveratrol for Breast Cancer Prevention and Therapy: Preclinical Evidence and Molecular Mechanisms. Semin Cancer Biol (2016), 40-41:209–32. 10.1016/j.semcancer.2015.11.001 26774195

[25] Mokni M Elkahoui S Limam F Amri M Aouani E. Effect of Resveratrol on Antioxidant Enzyme Activities in the Brain of Healthy Rat. Neurochem Res (2007) 32(6):981–7. 10.1007/s11064-006-9255-z 17401679

[26] Albani D Polito L Signorini A Forloni G. Neuroprotective Properties of Resveratrol in Different Neurodegenerative Disorders. BioFactors (2010) 36(5):370–6. 10.1002/biof.118 20848560

[27] Guo Y Dong S Cui X Feng Y Liu T Yin M. Resveratrol Alleviates MPTP-induced Motor Impairments and Pathological Changes by Autophagic Degradation of α-Synuclein Via SIRT1-Deacetylated Lc3. Mol Nutr Food Res (2016) 60(10):2161–75. 10.1002/mnfr.201600111 PMC6089356 27296520

[28] Gomes B Silva J Romeiro C Dos Santos S Rodrigues C Gonçalves P. Neuroprotective Mechanisms of Resveratrol in Alzheimer’s Disease: Role of SIRT1. Oxid Med Cell Longevity (2018) 2018:8152373. 10.1155/2018/8152373 PMC6232815 30510627

[29] Chen J Liu Q Wang Y Guo Y Xu X Huang P. Protective Effects of Resveratrol Liposomes on Mitochondria in Substantia Nigra Cells of Parkinsonized Rats. Ann Palliative Med (2021) 10(3):2458–68. 10.21037/apm-19-426 33549012

[30] Gawlik M Gawlik M Smaga I Filip M. Manganese Neurotoxicity and Protective Effects of Resveratrol and Quercetin in Preclinical Research. Pharmacol Rep (2017) 69(2):322–30. 10.1016/j.pharep.2016.11.011 28183032

[31] Li X Feng Y Wang X Truong D Wu Y. The Critical Role of SIRT1 in Parkinson’s Disease: Mechanism and Therapeutic Considerations. Aging Dis (2020) 11(6):1608–22. 10.14336/ad.2020.0216 PMC7673849 33269110

[32] Meadows S Chambers N Conti M Bossert S Tasber C Sheena E. Characterizing the Differential Roles of Striatal 5-HT Auto- and Hetero-Receptors in the Reduction of l-DOPA-Induced Dyskinesia. Exp Neurol (2017) 292:168–78. 10.1016/j.expneurol.2017.03.013 28342749

[33] Chotibut T Meadows S Kasanga E McInnis T Cantu M Bishop C. Ceftriaxone Reduces L-dopa-Induced Dyskinesia Severity in 6-Hydroxydopamine Parkinson’s Disease Model. Mov Disord Off J Mov Disord Soc (2017) 32(11):1547–56. 10.1002/mds.27077 PMC5681381 28631864

[34] Eskow K Gupta V Alam S Park J Bishop C. The Partial 5-HT(1A) Agonist Buspirone Reduces the Expression and Development of l-DOPA-Induced Dyskinesia in Rats and Improves l-DOPA Efficacy. Pharmacol Biochem, Behav (2007) 87(3):306–14. 10.1016/j.pbb.2007.05.002 17553556

[35] Rajput AH. Factors Predictive of the Development of Levodopa-Induced Dyskinesia and Wearing-Off in Parkinson’s Disease. Mov Disord (2013) 28(3):1064–71. 10.1002/mds.25364 23630119

[36] Mytilineou C Han S Cohen G. Toxic and Protective Effects of L-dopa on Mesencephalic Cell Cultures. J Neurochem (1993) 61(4):1470–8. 10.1111/j.1471-4159.1993.tb13642.x 8376999

[37] Fanni S Scheggi S Rossi F Tronci E Traccis F Stancampiano R. 5alpha-Reductase Inhibitors Dampen L-DOPA-Induced Dyskinesia Via Normalization of Dopamine D1-Receptor Signaling Pathway and D1-D3 Receptor Interaction. Neurobiol Dis (2019) 121:120–30. 10.1016/j.nbd.2018.09.018 30261284

[38] Dos-Santos-Pereira M da-Silva C Guimarães F Del-Bel E. Co-Administration of Cannabidiol and Capsazepine Reduces L-DOPA-Induced Dyskinesia in Mice: Possible Mechanism of Action. Neurobiol Dis (2016) 94:179–95. 10.1016/j.nbd.2016.06.013 27373843

[39] Park H Ryu Y Kim Y Park T Go J Hwang J. Gadd45β Ameliorates L-DOPA-Induced Dyskinesia in a Parkinson’s Disease Mouse Model. Neurobiol Dis (2016) 89:169–79. 10.1016/j.nbd.2016.02.013 26875664

[40] Mulas G Espa E Fenu S Spiga S Cossu G Pillai E. Differential Induction of Dyskinesia and Neuroinflammation by Pulsatile Versus Continuous l-DOPA Delivery in the 6-OHDA Model of Parkinson’s Disease. Exp Neurol (2016) 286:83–92. 10.1016/j.expneurol.2016.09.013 27697481

[41] Ahmad R Kochumon S Chandy B Shenouda S Koshy M Hasan A. Tnf-α Drives the CCL4 Expression in Human Monocytic Cells: Involvement of the SAPK/JNK and NF-κb Signaling Pathways. Cell Physiol Biochem (2019) 52(4):908–21. 10.33594/000000063 30964608

[42] Son M Wu J. Egg White Hydrolysate and Peptide Reverse Insulin Resistance Associated With Tumor Necrosis Factor-α (Tnf-α) Stimulated Mitogen-Activated Protein Kinase (MAPK) Pathway in Skeletal Muscle Cells. Eur J Nutr (2019) 58(5):1961–9. 10.1007/s00394-018-1753-7 PMC6647935 29955954

[43] Takeuchi H Jin S Wang J Zhang G Kawanokuchi J Kuno R. Tumor Necrosis Factor-Alpha Induces Neurotoxicity Via Glutamate Release From Hemichannels of Activated Microglia in an Autocrine Manner. J Biol Chem (2006) 281(30):21362–8. 10.1074/jbc.M600504200 16720574

[44] Mercurio A Adriani G Catalano A Carocci A Rao L Lentini G. A Mini-Review on Thalidomide: Chemistry, Mechanisms of Action, Therapeutic Potential and Anti-Angiogenic Properties in Multiple Myeloma. Curr Med Chem (2017) 24(25):2736–44. 10.2174/0929867324666170601074646 28571559

[45] Bortolanza M Padovan-Neto F Cavalcanti-Kiwiatkoski R Dos Santos-Pereira M Mitkovski M Raisman-Vozari R. Are Cyclooxygenase-2 and Nitric Oxide Involved in the Dyskinesia of Parkinson’s Disease Induced by L-DOPA? Philos Trans R Soc London Ser B Biol Sci (2015) 370:(1672). 10.1098/rstb.2014.0190 PMC4455759 26009769

[46] Del-Bel E Padovan-Neto F Bortolanza M Tumas V Aguiar A Raisman-Vozari R. Nitric Oxide, a New Player in L-DOPA-Induced Dyskinesia? Front biosci (2015) 7:168–92. 10.2741/e726 25553372

[47] Asanuma M Miyazaki I Murakami S Diaz-Corrales F Ogawa N. Striatal Astrocytes Act as a Reservoir for L-DOPA. PloS One (2014) 9(9):e106362. 10.1371/journal.pone.0106362 25188235 PMC4154692

[48] Inyushin M Huertas A Kucheryavykh Y Kucheryavykh L Tsydzik V Sanabria P. L-DOPA Uptake in Astrocytic Endfeet Enwrapping Blood Vessels in Rat Brain. Parkinson’s Dis (2012) 2012:321406. 10.1155/2012/321406 22888467 PMC3409556

[49] Tsai M Lee E. Characterization of L-DOPA Transport in Cultured Rat and Mouse Astrocytes. J Neurosci Res (1996) 43(4):490–5. 10.1002/(sici)1097-4547(19960215)43:4<490::Aid-jnr10>3.0.Co;2-6 8699535

[50] Youdim M. My Love With Monoamine Oxidase, Iron and Parkinson’s Disease. J Neural Transm Supplementum (2006) (71):5–9. 17447409

[51] Fonteles A Neves J Menezes A Pereira J Silva A Cunha R. Atp Signaling Controlling Dyskinesia Through P2X7 Receptors. Front Mol Neurosci (2020) 13:111. 10.3389/fnmol.2020.00111 32848592 PMC7427508

[52] Soliman M Mazzio E Soliman K. Levodopa Modulating Effects of Inducible Nitric Oxide Synthase and Reactive Oxygen Species in Glioma Cells. Life Sci (2002) 72(2):185–98. 10.1016/s0024-3205(02)02204-x 12417252

[53] Avila-Luna A Ríos C Gálvez-Rosas A Montes S Arias-Montaño J Bueno-Nava A. Chronic Administration of the Histamine H Receptor Agonist Immepip Decreases L-Dopa-Induced Dyskinesias in 6-Hydroxydopamine-Lesioned Rats. Psychopharmacology (2019) 236(6):1937–48. 10.1007/s00213-019-5182-y 30762089

