# Supplementary figures and images for: Resveratrol Alleviates Levodopa-Induced Dyskinesia in Rats
Source: Front Immunol. 2021 Jun 25;12:683577. doi: 10.3389/fimmu.2021.683577 (PMC8267475; doi:10.3389/fimmu.2021.683577)

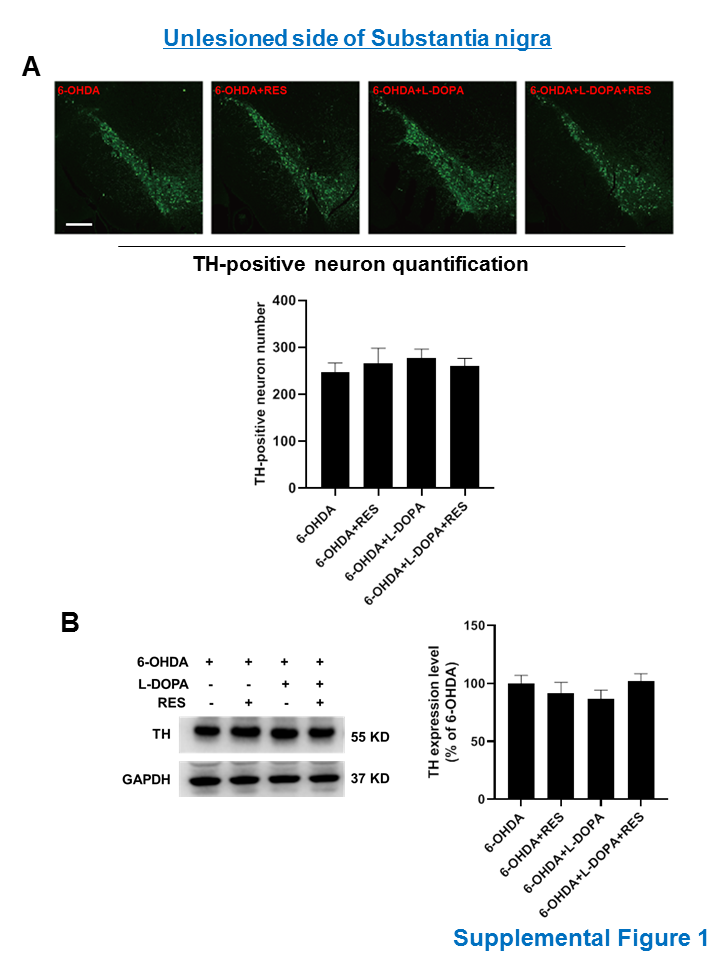

Supplement: Supplementary Figure 1 — Effects of RES and L-DOPA on DA neuronal survival in the unlesioned side of substantia nigra. After 42 days of L-DOPA treatment, rats were sacrificed and brains were collected, respectively. DA neurons in substantia nigra were recognized and immunostained with an anti-TH antibody. DA neuronal survival in the unlesioned side of substantia nigra was analyzed via the quantification of TH-positive neurons (A) and the expression of TH protein (B). Scale bar=100 µm. The densitometry values of TH protein were detected and normalized to GAPDH of the same group. Then, data were normalized and calculated as a percentage of each respective 6-OHDA group. Data were expressed as mean ± SEM from 5 rats. [file Image_1.tif]
